# SpliceImpactR maps alternative RNA processing events driving protein functional diversity

**DOI:** 10.1093/nar/gkag827

**Published:** 2026-08-17

**Authors:** Zachary Peters Wakefield, Ana Fiszbein

**Affiliations:** Bioinformatics Program, Faculty of Computing and Data Sciences, Boston University, Boston, MA 02215, United States; Biology Department, Boston University, Boston, MA 02215, United States; Bioinformatics Program, Faculty of Computing and Data Sciences, Boston University, Boston, MA 02215, United States; Biology Department, Boston University, Boston, MA 02215, United States

## Abstract

Alternative RNA processing is a key regulator of gene expression, shaping transcript and protein diversity essential for cell function. Yet, how distinct alternative RNA processing events alter protein function remains unclear. Here, we introduce SpliceImpactR—available as a Bioconductor package and interactive R Shiny application—an open-source framework that systematically identifies RNA isoform switches across the human transcriptome, including alternative first and last exons, exon skipping, intron retention, hybrid exons, and splice site variants, and predicts their impact on encoded proteins. Applying SpliceImpactR across all annotated human isoforms and 17 350 samples spanning 54 tissues, we find that intron retention and hybrid exons frequently shift protein-coding transcripts into non-coding isoforms. Strikingly, when both isoforms remain protein-coding, 87% of alternative RNA processing events produce substantial changes in protein sequence. Widespread frameshifts introduced by alternative splicing are often rescued by co-regulated downstream exons, uncovering a buffering mechanism that maintains protein integrity. Alternative last exons drive the most extensive structural changes, while alternative first exons emerge as the most efficient mechanism for reshaping tissue-specific protein domain architecture. We further found that alternative RNA processing events are widely co-regulated and undergo gradual, tissue-specific transitions rather than binary on/off switches. In the cerebellum, we found that alternative first exons progressively increase and activate nuclear localization signals, while in colon adenocarcinoma internal splicing events rewire key oncogenic protein interactions. Together, SpliceImpactR delivers a proteome-wide atlas of splicing regulation, establishing a powerful, accessible platform to decode how context-dependent RNA processing drives human proteomic and tissue diversity.

## Graphical Abstract

**Graphical Abstract:**
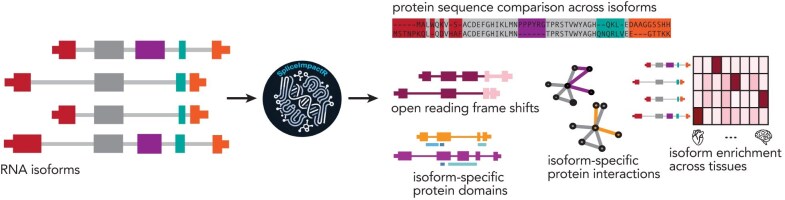

## Introduction

Early transcriptomic studies showed that most human genes generate multiple mRNA isoforms through alternative splicing of internal exons. More recently, however, alternative first and last exons have been identified as even greater contributors to transcript diversity [1]. Collectively, alternative RNA processing events impact nearly every aspect of gene function, from transcript subcellular localization and translational efficiency to protein domain architecture and post-translational modifications [2–5]. These changes can reshape protein structures, modulate post-translational modification sites, and rewire protein–protein interaction (PPI) networks [5, 6].

There are several major classes of alternative RNA processing events. Skipped exons (SE) are internal exons that are included in some transcripts and excluded in others. Retained introns (RI) are introns that remain in the mature transcript rather than being spliced out. Alternative 3′ and 5′ splice sites (A3SS and A5SS) cause variation at exon boundaries by shifting the precise splice junctions. Mutually exclusive exons (MXE) involve pairs of exons where only one is included in the mature mRNA. Alternative first exons (AFE) are regulated by different promoters, while alternative last exons (ALE) are associated with the use of alternative polyadenylation sites. Within first exons, alternative transcription start sites (TSS) differ in the first nucleotide transcribed. Within last exons, alternative transcription end sites (TES) refer to the use of different polyadenylation sites, leading to alternative 3′ untranslated regions. Together, these events dramatically increase isoform complexity and are tightly regulated.

The functional impact of alternative RNA processing in protein-coding genes is particularly pronounced in tissue-specific contexts. Distinct splicing patterns drive the production of specialized protein isoforms, enabling precise regulation of cellular processes [7–11]. For example, neurons exhibit extended 3′ untranslated regions and uniquely spliced exons critical for synaptic function, while immune cells rely on alternative splicing to diversify signaling pathways and adapt rapidly to pathogens [12, 13]. Dysregulation of these processes is increasingly linked to disease: in cancer, distinct isoforms can promote tumor progression, metastasis, and immune evasion [14–20]. Similarly, intron retention has been shown to be correlated with progression of Alzheimer’s disease [21]. However, the functional consequences of these changes remain poorly understood, particularly in complex physiological and disease contexts.

In recent years, numerous computational tools have been developed to predict the downstream functional consequences of alternative RNA processing. These tools enable the analysis of alternative splicing changes across conditions [22], the prediction of protein domains that are altered [23–26], or facilitate protein-feature-integrated PPI studies [9, 26, 27]. They can assess the evolutionary conservation of alternative RNA processing events [28], compare protein isoforms using long-read sequencing approaches [29], and visualize how isoform changes may impact transcript and protein sequence [30]. However, the functional consequences of individual RNA processing events on the proteome remain difficult to interpret.

Alternative RNA processing is not a series of isolated events but a highly coordinated process, where decisions at one stage can significantly influence downstream choices. For example, alternative first and alternative last exon usage are interlinked within a positional initiation-termination axis, where decisions at the 5′ end directly affect those at the 3′ end [31, 32]. Coupling also occurs between alternative splicing and 3′ end processing [33] as well as between AFE and alternatively spliced internal exons [34–36]. In fact, we recently discovered more than 100 000 human *hybrid exons* that can both serve as terminal or internal exons. Hybrid first exons (HFE) can act as first exons or internal exons, while hybrid last exons (HLE) act as internal or last exons in different transcripts [37]. Despite this well-documented co-regulation of different RNA processing events, no existing computational pipeline integrates all these aspects to predict the functional impact of isoform-specific changes at the gene level.

To address this systematically, here we developed SpliceImpactR, an integrative, open-source R package designed to unite exon-level information from RNA-seq data to reveal how differentially used alternative RNA processing events shape protein function. Unlike existing tools that analyze transcript-level isoform changes and rigid sets of protein consequences, SpliceImpactR directly interprets exon-level RNA processing events to predict their integrated protein consequences, including domains, motifs, frameshifts, and PPI rewiring, to predict gene-level functional impact in a single, efficient pipeline with built-in visualization. Running SpliceImpactR across all annotated human isoforms and 17 350 samples from 54 human tissues, we found that RI and hybrid exons drive the most significant shift toward non-protein-coding mRNAs, while ALE lead to the largest changes in protein primary sequences and domains, and alternative 5′ and 3′ splice sites introduce the most substantial frameshifts. Interestingly, we found that, across human tissues, AFE drive the largest relative changes in protein sequences resulting in tissue-specific protein domains, and vital protein interactions are modulated by alternative splicing events in colon adenocarcinoma. SpliceImpactR is available through Bioconductor’s repository of software https://bioconductor.org/packages/SpliceImpactR/ and through an R Shiny interface https://fiszbein-lab.shinyapps.io/SpliceImpactR_Studio/.

## Materials and methods

### SpliceImpactR pipeline

**Figure 1.**
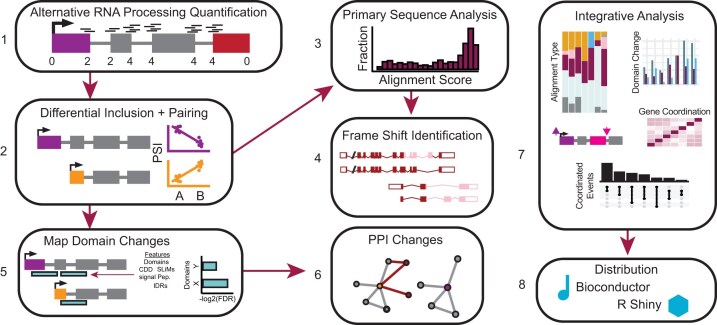
SpliceImpactR pipeline. (1) SpliceImpactR takes as input alternative RNA processing events described through inclusion and exclusion coordinates. (2) Significant differential RNA processing events are identified based on statistical testing and delta percent-spliced-in (ΔPSI) thresholds. Transcripts differing by at least one such event are grouped into matched pairs. (3) Primary amino acid sequence comparisons are performed to assess sequence similarity and classify transcript-level differences. (4) InterPro domain annotations are mapped to the protein products of these transcript pairs to detect localized domain changes. (5) Identified domain changes are aggregated to reveal global patterns. (6) Known domain–domain and domain–motif interactions from 3did, PPIDM, and ELM are integrated with BioGrid to allow for determination of whether alternative RNA processing events alter protein–protein interfaces. (7) A comprehensive integrative analysis is performed across all differential events to identify co-regulated splicing patterns and predict their aggregated effects on proteins. (8) SpliceImpactR is accessible through Bioconductor’s software ecosystem or through SpliceImpactR Studio, an R Shiny web interface implementation of the workflow.

SpliceImpactR is a self-contained and efficient pipeline, aimed to identify how alternative RNA processing events impact resulting proteins. The computational method does not require any software external to R after the initial alternative RNA processing quantification is performed. SpliceImpactR begins by importing all alternative RNA processing events across conditions (Step 1). It is built to allow a highly flexible input, requiring inclusion coordinates and exclusion coordinates for each form of an event. This allows for user-defined events or events directly from tools. Custom input functions detect changes in alternative RNA processing events, including AFE, HFE, RI, SE (often referred to as cassette exons), A5SS and A3SS, MXE, HLE, and ALE. It then performs differential inclusion analysis to identify condition-specific pairs of transcripts that differ in at least one alternative RNA processing event (Step 2). These differentially included events are mapped to transcript and protein annotations to evaluate their effect on coding potential, sequence similarity, and frameshifts (Step 3). SpliceImpactR then uses sets of protein features along with optional user-provided features to annotate domain-level changes (Step 4), assess domain usage shifts across conditions (Step 5), and integrate domain–domain interaction (DDI) data, domain–motif interaction data, and overall PPI to predict impacts on PPIs (Step 6). Finally, it conducts a global analysis to detect co-regulated splicing events and quantify the relative contribution of each alternative RNA processing type (Step 7). SpliceImpactR is available through Bioconductor and implemented as an R Shiny instance for maximal ease of use and interpretability (Step 8; see Fig. 1). Unlike existing tools that focus on individual features, SpliceImpactR integrates multiple functional layers within a single pipeline. SpliceImpactR supports both data.table-based and S4-compatible workflows, maximizing usability across different analysis preferences. SpliceImpactR’s vignettes provide an exploration of how to use the package: https://bioconductor.org/packages/release/bioc/vignettes/SpliceImpactR/inst/doc/SpliceImpactR.html.

### Alternative RNA processing identification and quantification

SpliceImpactR allows for input from any relevant source. The base functioning of the package requires alternative RNA processing events to be defined through sequences of inclusion and sequences of exclusion. We also allow for input at the transcript level—or input that already has differential inclusion metrics attached. We have specific custom functions built to work directly downstream of popular software, such as the hybrid-internal-term (HIT) index [37] and multivariate analysis of transcript splicing (rMATS) [38].

These tools are applied to short-read RNA-sequencing data to produce relative inclusion values for alternative RNA processing events. The relative inclusion value generated by these tools is a percent-spliced-in (PSI) value, calculated through relative junction and exon reads. PSI values are the reads assigned to the specific event (junction and/or exon-covering reads) relative to the other terminal exons or the exclusion form. This metric is calculated differently for various tools, though represents a very similar measure. A generalized form for the PSI of exon *i* in gene *g* is


\begin{eqnarray*}
\mathrm{ PS}{{\mathrm{ I}}_{ig}} = \ \frac{{{{I}_{ig}}}}{{{{I}_{ig}} + {{E}_{ig}}}},
\end{eqnarray*}

where ${{I}_{ig}}$ represents the number of reads associated with the inclusion of exon *i* from gene *g*. ${{E}_{ig}}$ represents the number of reads associated with the exclusion of exon *i* in gene *g*. These tools enabled the identification of alternative events such as AFE, A5SS, MXE, RI, SE, A3SS, and ALE. SpliceImpactR uses the PSI values to generate profiles for samples from each condition. The HIT index also classifies exons as internal or terminal, allowing for the analysis of hybrid first and last exons (HFE, HLE). Sample output from these tools is imported into R and collapsed to prepare for downstream analysis. Events are ultimately transformed into sets of included and excluded coordinates, where binary events (A3SS, A5SS, MXE, SE, RI) are split further into the inclusion forms and the exclusion forms. Terminal exon processing events are processed into a similar form requiring inclusion coordinates—however as these events aren’t inherently paired and are able to be used in more complex patterns, they are not separated into inclusion and exclusion forms. SpliceImpactR assumes input is given in 1-based coordinate system.

### Fetching annotations

SpliceImpactR fetches annotations from Gencode [39], extracting comprehensive gene annotations, transcript sequences, and peptide sequences. We fetch protein features from the set available from biomaRt (e.g. InterPro [40], SignalP [41]). We allow for other protein features to be accessed from biomaRt [42, 43], as well as user-provided features. We also fetch validated short linear motifs (SLiMs) from the Eukaryotic Linear Motif (ELM [44]) database. The user-provided features require at a minimum either a transcript or protein Ensembl ID and the coordinates within the transcript or protein where the feature is located. This is a flexible accession as any compatible versions from the human or mouse genomes are available (as they are available from Gencode and biomaRt).

### Condition comparison

Global RNA processing profiles of samples across conditions are compared using event counts and PSI values from HIT Index [37] and rMATS [38]. To normalize for depth, we must first calculate size factors in each sample. This is done using the same method as Anders and Huber (2012) [45]:


\begin{eqnarray*}
{{\hat{s}}_j} = \mathrm{media}{{\mathrm{ n}}_i}\frac{{{{k}_{ij}}}}{{{{{\left( {\mathop \prod \nolimits_{v = 1}^m {{k}_{iv}}} \right)}}^{1/m}}}},
\end{eqnarray*}

where ${{( {\mathop \prod \limits_{v = 1}^m {{k}_{iv}}} )}^{1/m}}$ is the geometric mean of reads covering exon *i* in all *m* samples and ${{k}_{ij}}$ are the reads covering exon *i* in sample *j*. One difference here is that the reads incorporated into the size factors stem from only the RNA processing event being analyzed. First and last exon processing events use junction-covering reads to calculate size factors instead of exon-covering reads. Event counts are normalized using the size factors to control for variations in sequencing and filtered for low expressed events. SpliceImpactR performs comparisons such as event count, distributions of event per gene, and cumulative distribution function of global PSI values are performed to identify shifts in macro splicing patterns across conditions. We use a Wilcoxon rank sum test to obtain a *P*-value for these count changes across conditions. We also generate visualizations and output statistics of how HIT Index values assigned to exons are changing—also extracting exons that are significantly changing using the Wilcoxon rank sum test followed by adjusting *P*-values to FDR.

### Differential analysis and pairing

Differential inclusion of alternative RNA processing is identified through the difference in average PSI value across experimental condition—or a delta PSI score. The pipeline excludes low-coverage events (defaulting to a threshold of 10 inclusion reads for each event) and those with a high number of missing values before applying a statistical model. Next, quality filtering is performed. In the cases of AFE, HFE, HLE, and ALE only exons that are annotated as first or last exons in at least one transcript are included for analysis by default. PSI values are recalculated using the updated filtered event information. After all filtering is done, the pipeline fills in information for events that are not present in all samples through assigning counts of inclusion of 0 and exclusion of the mean exclusion reads per event per sample. We then remove information relating to genes with solely psi values of 0 on a sample basis. Inclusion reads are extrapolated for AFE, ALE, HFE, and HLE from the difference in upstream and downstream junction reads for each exon. Exclusion reads for the same events are further calculated through the sum of the inclusion reads from all exons other than the given exon in each gene. We use the following statistical model for identifying differential usage of alternative RNA processing events. We model the PSI value and total count of reads through a quasibinomial generalized linear model with the experimental condition as a predictor. This allows for using PSI values directly and not having to adjust for exon length in the counts of inclusion or exclusion. Using the quasibinomial formulation ${{y}_i} = \mathrm{ PS}{{\mathrm{ I}}_i}\times{{e}_i}$, where the proportion $\mathrm{ PS}{{\mathrm{ I}}_i}$, the relative use of exon *i*, is equal to the inclusion reads of exon *i* (${{y}_i}$) divided by the sum of the inclusion and exclusion reads of exon *i (*${{e}_i}$*)*. Here, we model the reads of exon *i* as


\begin{eqnarray*}
{{y}_i}\ \sim \ \mathrm{Quasibinomial}\ \left( {{{p}_i},\ \theta } \right),
\end{eqnarray*}

\begin{eqnarray*}
{\mathrm{logit}}\left( {{{p}_i}} \right) = {{\beta }_t},
\end{eqnarray*}

Where ${{\beta }_t}$ accounts for the impact of experimental condition, $\theta $ is the dispersion parameter, and ${{p}_i}$ is the probability of reads covering exon *i*. This method circumvents the exon-length issue and serves as a computationally efficient approach for large-scale evaluations. The generalized linear model was implemented using glm in R with family of quasibinomial. We use the standard workflow for dispersion parameter estimation through the function. Here, we extract a *P*-value using an F-test to compare deviance and test for significance based on impact of experimental condition in nested models. Outlier values are identified using Cook’s distance and removed based on a user-specified threshold, with preset thresholds of 4/*n* (where *n* is the number of observations/sample) and 1. Cook’s distance outlier removal is only performed when the number of samples is ≥10. *P*-values generated through this method are adjusted for multiple hypotheses to generate a false discovery rate, with a default FDR of 0.05 being considered significant. Alternative RNA processing events are mapped to annotated exons using overlap of the query inclusion sequences and annotation exon. Exclusion coordinates are also mapped and require absence of overlap to allow a match to be retained. We prioritize protein-coding transcripts, transcript quality (transcript-support level), correct exon class (internal, terminal), and overlap length. Proteins and transcripts associated with each matched annotated exon were retrieved for subsequent analysis. For data with the possibility for combinations of more than two events (e.g. sourced from the HIT index), alternative events—and their associated transcripts and proteins—from the same gene with opposing inclusion preferences were paired for downstream analysis. In cases where more than two events from a gene showed differential inclusion, pairings were made in a pairwise manner between all combinations of upregulated and downregulated events. Data for events with strictly two forms per event (e.g. data from rMATS) are already paired, so no pairing operations are performed on it, and the alternative splicing forms are extracted from the coordinates supplied by each event ID.

### Primary sequence local analysis

We assess paired alternative RNA processing events’ transcripts for protein-coding status and classify as such if one or both transcripts are non-protein coding. The nucleotide sequences of the pairs of protein-coding transcripts containing paired alternative RNA processing events and the associated amino acid sequences were analyzed for sequence alignment using a NUC44 and BLOSUM62 scoring matrix. An alignment score is extracted to quantify sequence similarity—produced by taking the matching positions and dividing by the total non-gap sites. We use the R package pwalign for this global sequence alignment analysis [46]. We also compute transcript and protein length comparisons between the paired events.

Pairs that are both protein coding transcripts are further classified as having a frameshift or not having a frameshift. Frameshifts contain a reading frameshift between the overlapping coding regions in the exons involved in the alternative processing event, leading to the production of different amino acids from the same nucleotides. Frameshifts are identified by comparing reading frames relative to respective TSS at overlapping coding regions of each transcript. These overlapping coding regions are restricted to the exons containing the specific alternative splicing event being investigated, however AFE/HFE and ALE/HLE use the closest exons with overlapping coding regions. We identify downstream “rescues” by identifying where the reading frames may realign between the paired transcripts. We also identify whether the transcripts already had shifted frames upstream of the events to note when the event did not initiate the shift. The positional distribution of preferentially included exons (proximal versus distal) is also analyzed within AFE/ALE/HFE/HLE to detect potential global shifts in positional exon inclusion. The pipeline also performs an analysis of protein length, where it compares pairwise protein length. We use a Wilcoxon signed-rank test to identify whether there is a statistically significant shift in lengths between experimental conditions (*P*-value <.05 is considered significant). We also map the distribution of protein length changes in this output.

### Global proteomic analysis

We integrated protein-feature annotations and interaction data to quantify domain-level and PPI consequences of isoform switches. We build an exon-level domain table using annotations, where we allow for any biomaRt [42, 43] protein feature to be extracted. We also allow user-provided protein features to be added—only requiring a protein or transcript and the location in the sequence of the domain. This effectively allows for quick use of common queries, such as InterPro [40], PFAM [47], CDD [48], Mobidb [49], Signalp [41], TMHMM [50], and Ncoils [51]. For each event, we identified domains present due to the alternative exons relevant to the event. We also pull SLiMs from the Eukaryotic Linear Motif (ELM) [44] database of experimentally validated motif occurrences. To test enrichment of domains among foreground events versus a background, we built a background set of transcripts and matched domains to them. This background can either be sourced from annotations, a user-given list of transcripts, or the HIT Index output supplied. From there, we identified all possible pairs of transcripts and made a background set of possible domain changes, identifying possible domain changes across the transcripts. A hypergeometric enrichment test was used to identify the globally enriched domains, using the foreground from the differentially included set and the background we built. The *P*-values from the hypergeometric enrichment test are adjusted for multiple hypothesis testing to calculate a false discovery rate, with FDR < 0.05 being considered significant. The tool allows for the removal of repeating domains, which change multiple times between one pair of proteins to not overrepresent them in the enrichment plot. We also generate plot output of the enriched domains.

### Protein–protein interaction changes

A PPI network spanning proteins from over 16 000 genes was built using BioGrid. Using the domains identified in each protein, Protein–Protein Interaction Domain Miner’s (PPIDM) [44, 52] set of DDI was used to identify modulated PPI by DDI. We used the silver and gold sets of PPIDM interactions, along with the entirety of the gold standard set of structurally validated DDI from 3did [53]. Similarly, ELM’s set of domain–motif interactions was used to identify PPI modulated by DMIs. Using instances of domains from PFAM and SLiMs from ELM, we can use this rewired PPI network to identify when altered protein features drive changes in interactions. This network contains ~85 000 interactions modulated by DDI and 5000 interactions modulated by domain–motif interactions.

### Holistic analysis

To integrate information across multiple alternative RNA processing events, we incorporated a holistic analysis comparing the use of each event. We calculate relative usage of each event by dividing the number of significantly changing events by the total number of events identified in all the samples. We assess how events co-occur within the same genes from different event types. And further, we show how alignment scores, alignment classifications, and domain changes compare across event type. SpliceImpactR also allows for the probing of a specific event to visualize changes in PSI value and protein/transcript changes. The workflow similarly contains genome browser-like functionality for visualizing protein feature changes across transcripts.

### Gene set enrichment in SpliceImpactR

For our gene set enrichment analysis functionality within SpliceImpactR, we used the R package clusterProfiler [54] and hypeR [55] to process enrichment across Gene ontology, KEGG, Hallmark, and Reactome gene sets.

### Analyses across human isoforms annotated in the reference genome

To globally identify transcript swaps due to alternative RNA processing events in the annotated genome, we identified every alternative RNA processing event from the previous nine categories mentioned, along with alternative transcription start and end sites (aTSS, aTES). aTSS and aTES were defined as overlapping terminal exons with different terminal ends—differentiated from ALE and AFE (and HFE/HLE), which were defined to necessarily require nonoverlap between terminal exons. Using Ensembl annotation from biomaRt [42, 43], transcripts were kept if they were part of Ensembl’s transcript support level 1, Ensembl canonical, or Gencode basic transcript sets. Single exon transcripts were filtered out from analysis. Transcripts were also removed if they did not contain complete 3′ and 5′ ends. No isoforms were designated as primary; instead, transcripts were matched pairwise within each gene. Alternative RNA processing events were explicitly identified, with unique requirements for each event type. All exons containing alternative splicing were required to only overlap one exon, except for IR. SE and MXE were not limited to examples with just one exon involved in the exclusive inclusion. Transcript pairs that were both non-protein-coding were filtered out from further analysis. The sets of paired transcripts were used as input for adjusted functionality from SpliceImpactR.

We used the procedure from SpliceImpactR to identify frameshifts and rescues due to annotated RNA processing events. To identify when alternative RNA processing events were co-occurring, we extracted the set of transcript pairs for each event type and used a Dice–Sørensen coefficient to compute similarity metrics in a pairwise manner between the sets:


\begin{eqnarray*}
\mathrm{ DSC}\left( {{{e}_1},{{e}_2}} \right) = \frac{{2\left| {{{c}_{{{e}_1}}} \cap {{c}_{{{e}_2}}}} \right|}}{{|{{c}_{{{e}_1}}}\left| + \right|{{c}_{{{e}_2}}}|}},
\end{eqnarray*}

where we take two times the length of the intersect between the two sets divided by the sum of the lengths of each of the sets. This similarity metric was used because it accounts for large differences in sizes, such as the difference between the number of MXE and the number of aTSS. We also extracted the counts of events that were co-occurring. Looking at how frameshifts and rescues pair alternative RNA processing events, we looked at where the rescues occurred and identified what event type the respective exons belong to. We then standardized this by frameshift event count. We used procedures from SpliceImpactR to identify domains and enriched domains. The background set was adjusted to be composed from domains changing across any pair of transcripts from the annotated genome. The enrichment analysis here required the domains to be present in at least five genes to ensure one gene with a high number of transcripts wouldn’t be responsible for the domain’s enrichment, given the pairwise approach. We used dcGOR to identify enriched gene ontology biological processes associated with the enriched changing domains [56] (FDR < .05). To identify whether similar domains were changing across event type, we used the Dice–Sørensen index to generate a similarity metric between the domains changed across event type in a pairwise manner.

### Analyses across human samples and tissues from Genotype Tissue Expression

Aligned Genotype Tissue Expression (GTEx) version 8 RNA-seq data from 17 350 samples across 54 human tissues were downloaded from the AnVIL repository using the gen3-client and checked to be intact with samtools quick-check [57]. We use Gencode v45 for this analysis. The data were then processed with the HIT index pipeline using parameters readnum 10, overlap 15, and bootstrap 5. For added confidence, all hybrid exon calls were required to be assigned a hybrid probability of at least 0.8 by the pipeline’s generative model (see Fiszbein *et al*., [37]), and AFE and ALE PSI calculations were restricted to exons that were annotated as terminal exons. The aligned data were also processed with rMATS in statoff mode. Using these samples, we used SpliceImpactR on a pairwise basis across each tissue type. We set a threshold of 0.15 for delta psi and 0.05 for FDR. This resulted in 1431 sets of results containing analysis in all nine alternative RNA processing event types.

We used the pheatmap package [58] in R for each hierarchical clustering with method set as UPGMA. To compare the global changes between tissues, we calculated the average number of paired significantly differentially included alternative RNA processing events across all event types. To compare the alternative RNA processing events between blood, brain, and heart, we used the overlap in genes with changing events between tissue type, calculated using a Jaccard index, and used UPGMA for hierarchical clustering in pheatmaps’s function. To identify protein domains changing between one tissue and all other tissues, we extracted the list of domains upregulated for the given tissue and assigned all downregulated domains to the other tissue. Filtering out domains that were not commonly changing (with a count below the mean count of each domain), we assigned a value of 1 or 0 for inclusion or exclusion in each tissue type. Using the same UPGMA approach, we performed hierarchical clustering. To calculate relative change in inclusion, we took the difference between the mean number of times a domain was present in each tissue and all other tissues.

To identify the relative use of each event type, we first extract the total number of events identified after the previously established filtering for quality of events. We then use the number of significantly changing paired events to generate a relative use metric for each event type. For all gene set enrichment analyses, we used the R package hypeR [55] to search for enrichment in various gene sets (Gene ontology, KEGG, Hallmark) using hypergeometric method [55]. To identify enriched gene ontology domains in each event type, we calculated the log_2_ enrichment of each term in each event. This was done through accessing the GO terms from InterPro2GO [59] and calculating the log_2_ of the relative frequency of each GO term in the foreground (domains changing due to significantly differential events) and background (all possible domain changes due to changes in transcripts). To identify how nuclear localization signal (NLS) inclusion is changing due to alternative RNA processing, we used 17 NLS consensus sequences extracted from Lu *et al*. [60]. We then used MEME-suite’s FIMO tool [61] to scan the amino acid sequences from two sets of proteins for these motifs. The first of these sets is sourced from annotation analysis, all the proteins containing an AFE between them. The second set contains the proteins associated with the AFE that are significantly changing between cerebellum and left-ventricle tissues from GTEx. If within a given pair, only one of the proteins returned a significant match for an NLS motif (*q*-value < 0.05), this was marked as a change in inclusion of an NLS.

Validation of coordinated alternative splicing events was done through the use of 88 long-read RNA-sequencing samples from GTEx spanning 14 tissues and cell lines [62]. The transcripts from these samples were quantified using FLAIR [63] and normalized to TPM. Presence of transcripts in the long-read dataset is calculated as present in more than one sample with positive TPM.

### Colon adenocarcinoma analysis

We used RNA-seq data from 23 patients with tumor and adjacent normal matched samples from the TCGA (phs000178.v11.p8) COAD project from release v36. We used rMATS [38] and the HIT index [37] with the same set of parameters as in the application to GTEx data to extract alternative RNA processing events. We used transcript quantification extracted using RSEM [64] data from UCSC’s Toil RNAseq Recomputed Compendium [65] set of TCGA samples from Gencode hg38 v23 to generate results from IsoformSwitchAnalyzer [24, 66] and tappAS [25]. These tools were used with their default parameters, with IsoformSwitchAnalyzer’s dIF threshold being set to 0.20. The threshold of delta PSI (and dIF) was increased from 0.2 to 0.45 and the overlap from the tool of focus in the other tools was calculated. Protein consequences were compared through looking at whether the protein domains identified as changing by SpliceImpactR were the same or a subset of the other tools where events were found as differentially included by at least two of the three tools. We compared the PPI changes between a pair of isoforms found by SpliceImpactR to the PPI changes in the same pair found by DIGGER for comparison. To test interactions between peptides, we used ColabFold [67] (version 1.5.5, alphafold2-multimer_v3) with default settings other than selection of calc_extra_ptm, 6 recycles, 5 num_models, and pair_mode set to paired. We then used PyMOL [68] to visualize the predicted interaction and structures, along with identify the residues associated with the relevant interaction. For validation of the AFE identified by the HIT index and further found as significantly changing by SpliceImpactR, we used FANTOM5’s [69, 70] set of hg38 reprocessed CAGE peaks in human tissue. We scanned for overlap between the AFE coordinates across various windows. For gene set enrichment, we used ClusterProfiler [54] to probe for enrichment in MSigDB’s Hallmark geneset [71].

## Results

### Alternative RNA processing has widespread impact on protein primary sequences

**Figure 2.**
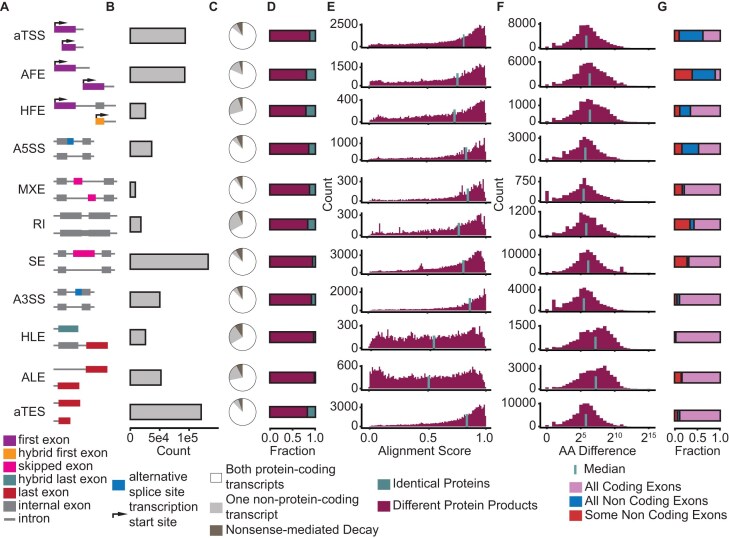
The widespread impact of alternative RNA processing on protein primary sequences. (**A**) Diagrams of the 11 forms of alternative RNA processing considered: aTSS (alternative transcription start sites), AFE (alternative first exons), HFE (hybrid first exons), A5SS (alternative 5′ splice sites), MXE (mutually exclusive exons), RI (retained introns), SE (skipped exons), A3SS (alternative 3′ splice sites), HLE (hybrid last exons), ALE (alternative last exons), and aTES (alternative transcription end sites). (**B**) Count of transcript pairs that differ in a given event among 171 397 annotated isoforms. (**C**) Fraction of transcript pairs differing in a given event that change protein-coding status, across NMD, no valid ORF, or both protein-coding. (**D**) Fraction of paired transcripts differing in a given event that result in either identical proteins or different proteins, when both transcripts are protein-coding. (**E**) Distribution of primary sequence similarity between protein isoforms encoded by each transcript differing in a given alternative RNA processing event. Alignment score is calculated through sequence alignment using BLOSUM62 [# matched amino acids/# total (non-gap) amino acids]. Vertical line shows the median alignment score. (**F**) Distribution of changes in protein length (log₂ scale) associated with each alternative RNA processing event type. The vertical line indicates the median length change for each event. (**G**) Proportion of alternative RNA processing events based on the genomic location of involved exons—classified as entirely within coding regions (“all coding exons”), entirely outside coding regions (“all non-coding exons”), or a mix of coding and non-coding regions (“some non-coding exons”).

We first used SpliceImpactR to explore the full spectrum of protein diversity generated by all forms of alternative RNA processing annotated in the human transcriptome. Using the full set of 171 397 isoforms annotated in the Gencode’s v45 annotation, we identified all transcript pairs with at least one difference in any of the 11 alternative RNA processing events considered (Fig. 2A), resulting in 654 444 paired transcripts. Notably, SEs were the most prevalent alternative processing event, differentially included in 132 100 transcript pairs, followed by aTES, aTSS, and AFEs. In contrast, MXE and RI were the least common, occurring in 8617–18 044 transcript pairs (Fig. 2B). We then analyzed which of these alternative processing events disrupt the protein-producing potential of their transcripts. To distinguish different mechanisms of protein-production disruption, we differentiate transcripts as either lacking a valid ORF or as containing premature termination codons (PTCs) predicted to trigger nonsense-mediated decay (NMD). We found that nearly 30% of RIs cause their transcripts to be predicted for NMD or lose an identifiable open reading frame when retained. Similarly, hybrid exons—whether used as terminal or internal exons—are strongly associated with protein-coding status changes, with 23% of cases shifting from a protein-coding to a non-coding transcript (Fig. 2C). Alternative first and last exon usage can also often lead to the loss of functional protein-coding transcripts. Interestingly, most transcript pairs containing RI, hybrid exons, or alternative first and last exons change the functional potential of transcripts through disrupting ORFs, rather than introducing a PTC to lead to NMD. However, SEs, MXEs, alternative splice sites, aTSSs, and aTESs rarely lead to changes in functional protein product outcomes. When these events do alter transcript functional potential, they show a higher relative ratio of NMD-targeted to loss-of-ORF transcripts than other event types (Fig. 2C). Strikingly, when alternative RNA processing events do not alter the protein-coding status of transcripts, we found that 87% still lead to changes in the resulting protein sequences (Fig. 2D). This indicates that only about 1 in 10 alternative RNA processing events in protein-coding genes will leave the encoded protein unchanged. Despite not being the most frequent alternative RNA processing events, ALEs and HLEs appear to drive the most significant changes in protein sequences with only ∼3% maintaining identical protein sequence (Fig. 2D). Paired proteins that differ in the usage of an ALE have the lowest median alignment score (0.51, Fig. 2E) and the largest change in protein length, with a median change of 145 amino acids (Fig. 2F). One of the most striking examples is the TTN gene, where the use of an upstream ALE shortens the transcript by >98 kilobases (Supplementary Fig. S1A). AFEs and HFEs rank next in driving substantial changes in protein sequences, with median alignment scores of 0.76 and 0.73 and a median change of 78 and 80 amino acids, respectively (Fig. 2E and F). This indicates that alternative changes at the 3′ end of transcripts have the greatest impact on protein sequence, followed by changes at the 5′ end, while alternative RNA processing events occurring within gene bodies result in smaller changes.

Interestingly, all types of RNA processing events include some cases that do not alter the final protein product. This is particularly common in AFEs and aTSS as they are frequently located in non-coding regions (Fig. 2G). When events occur within coding regions, we mostly observe intuitive cases where changes affect the resulting proteins, such as an AFE in *CHRAC1*, an MXE in *UBE2C*, and an A3SS in *C16orf92* where each form has distinct coding nucleotides (Supplementary Fig. S1B). However, we identified ∼3000 unexpected cases where changes within coding regions do not alter the resulting protein. These are often represented by short identical coding sequences, such as in the AFE of *MRM2* and the RI of *SPTLC1*. In *DBMT1*, a complex genomic rearrangement involving repeating sequences results in MXEs that encode identical protein sequence (Supplementary Fig. S1C).

### Co-regulation of alternative RNA processing rescues coding frameshifts

**Figure 3.**
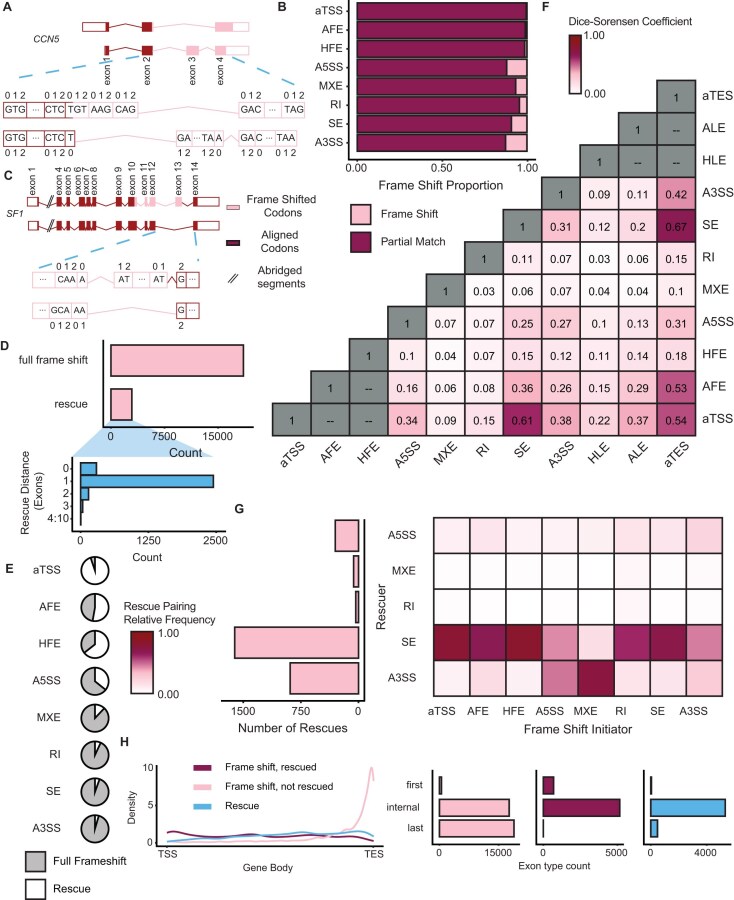
Analysis of frameshift impacts across annotated human protein-coding transcripts. (**A**) Example from the *CCN5* gene illustrating a frameshift initiated by an A5SS in exon 2, which introduces an 8-base extension at the downstream end of exon 1. This shift propagates through exon 4, altering the reading frame of all downstream overlapping exons. (**B**) Proportion of protein-coding transcript pairs affected by frameshifts. (**C**) Example of a rescued frameshift in *SF1*. An A5SS in exon 10 causes a frameshift, which is then restored by the skipping of exon 13 (SE), realigning the downstream reading frame. (**D**) Total number of observed full frameshifts and frameshift rescues. Inset shows the distribution of rescue distances (in number of exons) between the initiating and rescuing events. (**E**) Proportion of frameshift-initiating events that are rescued, grouped by event type. (**F**) Dice–Sørensen coefficient measuring similarity among transcript sets for each alternative RNA processing event. Cases where one event is a subset of another (e.g. HFE within AFE) or perfect overlap are grayed out. (**G**) (Left) Count of rescue events grouped by alternative RNA processing type. (Right) Heatmap showing the proportion of rescuing events corresponding to each type of initiating frameshift event (normalized by column). (**H**) (Left) Density plots showing the distribution along the gene body of initiating frameshift events that are rescued, rescuing events, and unrescued frameshifts. (Right) Categorization of event-associated exons as first, internal, or last exons. This categorization refers to the exon type where the first frameshifted bases are seen in the transcript pairs.

Alternative RNA processing often leads to significant protein sequence changes by introducing frameshifts within coding regions. This commonly occurs when SE or alternative splice sites include or exclude nucleotides not a multiple of three. For instance, in *CCN5*, an A5SS extends exon 2 by eight nucleotides, shifting the reading frame in exon 4 and producing a completely different protein sequence (Fig. 3A). Such frameshifts alter how nucleotides are read, generating different codons, different amino acids, and ultimately completely distinct protein products. We found these shifts mainly stem from internal splicing events, with A3SS and A5SS accounting for frameshifts in roughly 14% of transcript pairs (Fig. 3B). We observed thousands of cases where an alternative processing event induces a frameshift that is corrected by a downstream event. For instance, in SF1, exon 10’s A5SS initiates a frameshift that continues across three exons until an SE event at exon 13 realigns the reading frame for the remaining exons (Fig. 3C). This type of event effectively replaces a whole section of the protein with a different amino acid sequence while preserving the terminal ends of the protein. Notably, we observed frame rescues in ~13.6% of all frameshift cases with almost 1 in 10 rescued within the same exon (Fig. 3D). An example is TRAPPC2L, where an aTSS induces a frameshift, and an A5SS in the same exon restores the original reading frame (Supplementary Fig. S2A). Beyond same-exon rescues, most frameshift rescues occur in the following exon, but some are observed up to 10 exons downstream (Fig. 3D). Frameshifts arising from alternative initiation events, such as aTSS, AFE, and HFE, are largely corrected, whereas those resulting from internal splicing events often remain unrescued (Fig. 3E).

To investigate which alternative RNA processing events commonly rescue others, we first analyzed patterns of co-occurrence across transcript pairs. We found that transcripts differing by at least one RNA processing event typically differ by three, indicating frequent combinatorial regulation (Supplementary Fig. S2B). Strong associations emerged between internal splicing events and alternative transcript starts and ends (Fig. 3F). This is consistent with previous work showing functional coupling between alternative first and internal or last exons^29,30,33^. In fact, the most common combinations involved aTSS, SEs, and aTES (Supplementary Fig. S2C). Given these patterns, it is not surprising that SEs are the most frequent rescuers of frameshifts, often compensating for disruptions introduced by aTSS, AFEs, or HFE (Fig. 3G).

Mapping frameshifts and rescues along the gene body show that internal exon frameshifts decline toward the 3′ end of genes, while rescue events increase (Fig. 3H). However, a significant proportion of unrescued frameshifts are found toward the end of the gene body, with approximately half occurring in the last exon of a transcript (Fig. 3H). Many of these cases involve alternative processing events introducing a frameshift only in the final exon— such as in *PIGV*, where an A3SS extends the final exon by 11 nucleotides (Supplementary Fig. S2D). These observations indicate that while frameshifts are a powerful source of proteome diversity, frame rescues can fine-tune this diversity by partially restoring the original reading-frame.

### Functional domain changes rewire protein–protein interactions

**Figure 4.**
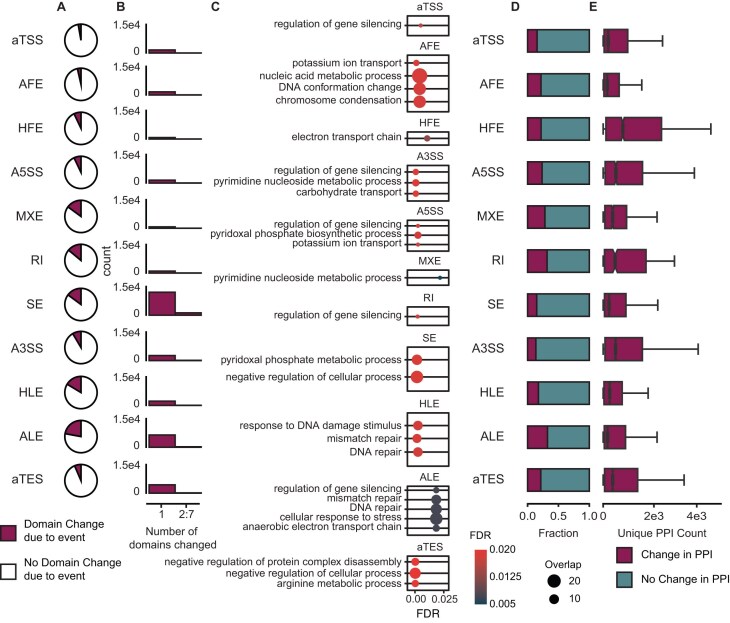
Functional domain changes induced by alternative RNA processing rewire PPI networks. (**A**) Proportion of transcript pairs for each alternative RNA processing event type that exhibit changes in InterPro-defined protein domains directly resulting from the alternative event. Domain changes are identified by overlapping the genomic coordinates of alternative events with annotated domain regions. (**B**) Number of domain changes per transcript pair for each event type, categorized as either a single domain change or two or more domain changes. (**C**) Gene ontology biological process enrichment of protein domains altered by each type of alternative RNA processing event, computed using the dcEnrichment() function from the dcGOR R package. Terms with FDR < 0.05 are considered significantly enriched. (**D**) Proportion of transcript pairs with each alternative RNA processing event type that result in rewiring of PPI networks. Rewiring is defined as the gain or loss of at least one DDI, based on the 3did database and domain presence in all human isoforms. (**E**) Distribution of the number of novel interactors gained per isoform within transcript pairs that exhibit PPI rewiring.

To determine which alternative RNA processing events directly impact protein function, we focused on events where both transcript variants remain protein-coding. Using the R package biomaRt [42, 43], we identified protein domains for all transcript pairs, mapping InterPro domains [40] to each peptide sequence, and identified cases where an alternative splicing event directly resulted in the addition or removal of a domain. Notably, we found that all types of alternative RNA processing events influence protein domain composition to some extent. ALEs have the largest impact on protein domains, with >21% directly resulting in changes to functional protein domain content (Fig. 4A). In contrast, aTSS have the lowest impact, with only ∼1.7% affecting a protein domain (Fig. 4A). Generally, when an alternative RNA processing event alters protein domains, it affects only one. However, instances exist across all event types where multiple domains are altered (Fig. 4B), with the highest recorded case involving changes in up to seven domains. For example, the use of a distal A5SS in exon 4 of the *MSH6* gene results in the inclusion of three additional protein domains essential for DNA mismatch repair (Supplementary Fig. S3A). Interestingly, protein domains affected by alternative RNA processing are predominantly linked to gene silencing regulation (Fig. 4C). AFE-driven domain changes are uniquely enriched for functions related to DNA conformation and chromosome condensation, while those arising from HLE and ALE events are specifically associated with DNA repair (Fig. 4C). When analyzing the domain composition across all isoform-altered proteins, we found frequent disruptions in zinc finger domains—particularly C2H2-type and RING-type—which are known to have a wide array of functions including mediation of protein, RNA, and DNA interactions (Supplementary Fig. S3B). This pattern suggests that alternative RNA processing may preferentially remodel interaction interfaces between proteins. To investigate this, we leveraged PPIDM’s DDI database (filtered to just contain the gold and silver-standard set of interactions) and ELM’s set of motif-domain interactions to predict how such domain alterations could rewire PPI networks. Among all event types, ALE and RI change the greatest proportion of protein interaction networks, with 32% of ALE changing at least one interactor (Fig. 4D). However, PPI networks rewired by HFE result in the largest change of novel interactors, with a median addition of 422 interactors per rewired network (Fig. 4E). This indicates that alternative RNA processing events significantly alter protein domain composition, and PPIs with ALE driving the largest changes.

Taken together, our analyses of human annotated isoforms highlight distinct roles for different alternative RNA processing events in shaping protein diversity. RI are the main contributors to isoforms with no identifiable open reading frame, whereas ALE and HLE events drive the most substantial alterations in protein sequences. Notably, ALE events also stand out as major drivers of changes in PPI networks. In contrast, SE, though the most common splicing event, have a relatively minor impact on protein diversity.

### Alternative RNA processing has widespread functional consequences across human tissues

**Figure 5.**
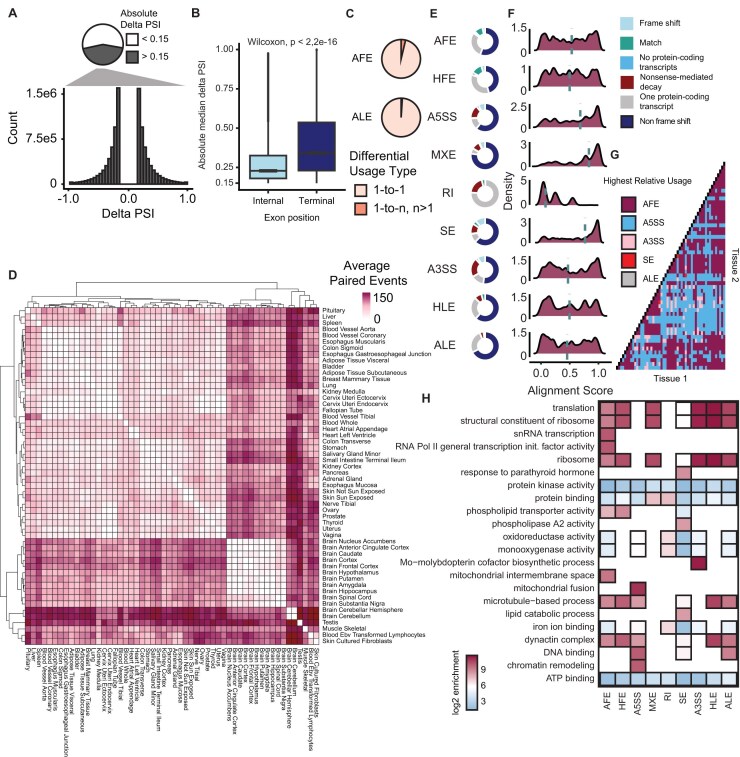
Alternative RNA processing has widespread functional consequences across human tissues. (**A**) *Top:* Proportion of alternative RNA processing events exceeding the significance threshold (|ΔPSI| > 0.15) across all GTEx tissue pairwise comparisons. *Bottom:* Distribution of ΔPSI values within the significant subset. Values were computed using pairwise runs of SpliceImpactR across GTEx tissues. (**B**) Distribution of absolute median delta psi scores split across terminal (AFE, HFE, HLE, ALE) and internal (A5SS, A3SS, MXE, SE, RI) exons. (**C**) Proportion of binary 1-to-1 changes in AFE and ALE versus more complex patterns involving three or more exons. (**D**) Hierarchical clustering (UPGMA) of the mean count of significantly changing events (|ΔPSI| > 0.15, FDR < 0.05) where both isoforms are protein-coding, calculated between each tissue pair using SpliceImpactR. (**E**) Proportion of protein-production potential combinations (e.g. coding-to-coding, coding-to-NMD, coding-to-noncoding) across significantly changing events. (**F**) Density of primary protein sequence similarity between isoforms from each event. Alignment scores were calculated using BLOSUM62 as [# matched amino acids/# total (non-gap) amino acids]. Dashed line indicates the median score per event type. (**G**) Heatmap showing the proportion of significantly changing events across tissues for each alternative RNA processing type, calculated as (# significant changes/total observed events). (**H**) Heatmap showing the log_2_ enrichment of gene ontology extracted from InterPro2GO. Top three GO categories selected for each alternative RNA processing event by most enriched relative to other event types.

To explore how the proteome-level impacts of alternative RNA processing shape tissue identity, we analyzed 17 350 samples from 54 human tissues using data from the Genotype-Tissue Expression (GTEx) project. Using SpliceImpactR in a pairwise fashion, we identified functionally relevant RNA processing changes across tissues. We found that 44% of all annotated alternative RNA processing events exhibit significant changes across the human tissues analyzed, indicating that the genome actively leverages a substantial portion of its splicing potential to drive tissue-specific regulation (Fig. 5A). Among these tissue-specific events, the median absolute change in percent spliced-in (ΔPSI) was 0.24, with just 1.2% exceeding a ΔPSI of 0.9 across tissues (Fig. 5A). These findings indicate that alternative RNA processing is rarely governed by binary on/off switches but instead exhibits a graded, fine-tuned regulation across tissues. We found that terminal exons, first and last exons, change significantly more in magnitude compared to internal exons (Fig. 5B) and that most of their switches are simple, with 97% involving only two exons rather than complex patterns with three or more exons (Fig. 5C). We found that genes showing significant tissue-specific changes in alternative RNA processing were enriched in processes involving localization, cell morphogenesis, cytoskeleton organization, and cell growth (Supplementary Fig. S4A). Among them, genes with the highest number of RNA processing events changing across tissues, including TPM1, have well-documented roles in tissue-specific isoform expression (Supplementary Fig. S4B) [72–76]. We next clustered human tissues based on the number of significantly differential alternative RNA processing events across nine event types, excluding aTSS and aTES, which are difficult to robustly detect using RNA-seq alone. As anticipated, brain tissues clustered closely and were distinct from other tissue types. Similarly, tissues with related developmental or functional origins—such as blood, cervix, and heart—also grouped together (Fig. 5D). These results confirm that tissues with shared characteristics exhibit similar isoform usage patterns and demonstrate that SpliceImpactR effectively captures biologically meaningful alternative RNA processing variation across tissues.

Similar to our findings from annotated genome-wide transcript comparisons, we found that RIs primarily influence the presence of a valid open reading frame or inclusion of a PTC (Fig. 5E), while significant changes in ALE, A5SS, and HLE usage across tissues rarely resulted in identical proteins (Supplementary Fig. S4C). We found that in A5SS, SE, and A3SS, the cases where an event drives a change in production of a protein, NMD-targeted transcripts, and lack-of-ORF transcripts occur at similar rates. This is in contrast with other alternative RNA processing events, where lack-of-ORF transcripts are present at much higher rates than NMD-targeted transcripts (Fig. 5E).

When both transcript variants remained protein-coding, terminal exons, HFEs, and RIs led to the most pronounced changes in protein sequence similarity (Fig. 5F). Interestingly, we found frameshifts and rescues across all RNA processing types, except for RI. SE caused the highest absolute number of frameshifts, averaging 115 per tissue, though <5% were rescued (Supplementary Fig. S4D). AFE had the highest proportion of rescued frameshifts, with a median rescue rate of just over 11% (Supplementary Fig. S4D). Consistently with what we observed in annotated isoforms, downstream SE are the most frequent rescuers of upstream frameshifts caused by any alternative RNA processing event (Supplementary Fig. S4E). Focusing on frame shift/rescue pairs identified between skeletal muscle tissue and all other tissues, we found that the vast majority of the significantly changing transcripts we inferred from short-read are indeed expressed as predicted when analyzing long-read sequencing samples (Supplementary Fig. S4F). Although SE account for the highest absolute number of protein sequence changes, we found that AFE are proportionally more likely to result in protein-altering outcomes relative to the total number of significantly changing transcript pairs across human tissues (Fig. 5G). This suggests that AFE are the most efficiently used mechanism for altering protein sequences across tissues, showing the strongest impact in 47% of all tissue pair comparisons.

Interestingly, alternative splice sites (A5SS, A3SS) result in the largest median changes to protein domains (Supplementary Fig. S4G), with ALE driving the most substantial rewiring of PPI networks across tissues (Supplementary Fig. S4H). RNA recognition motifs were among the top domains changed by the majority of event types and C2H2-type zinc finger domains were uniquely associated with changes in ALE (Supplementary Fig. S4I). While most alternative RNA processing events changing across human tissues modify protein domains associated with translation, AFEs are uniquely enriched for changing domains associated with transcription initiation (Fig. 5H).

### Functional consequences of alternative splicing in cerebellum reveals unique tissue-specific behavior

**Figure 6.**
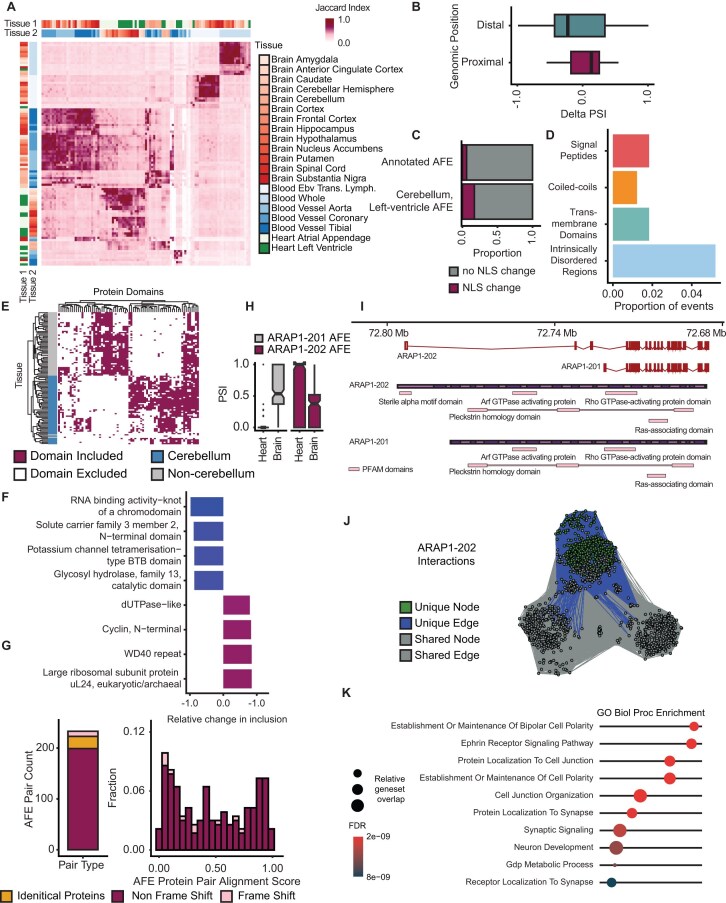
Functional consequences of alternative splicing in the cerebellum reveal distinct tissue-specific signatures. (**A**) UPGMA hierarchical clustering based on Jaccard indices of overlapping genes with significantly changed AFE events (|ΔPSI| > 0.15, FDR < 0.05) across blood, brain, and heart tissues. (**B**) Genomic distribution of cerebellum-enriched AFEs relative to left-ventricle-enriched AFEs. (**C**) NLS were identified using 17 consensus motifs from Lu *et al*. [60] and scanned using FIMO (MEME-suite). Transcript pairs with significant NLS motif changes (*q* < 0.05) were marked as showing altered NLS presence. (**D**) Proportion of AFE driving changes in coiled-coils (ncoils), transmembrane domains (tmhmm), signal peptides (signalp), and intrinsically disordered domains (mobidblite) between cerebellum and left-ventricle. (**E**) Hierarchical clustering (UPGMA) of commonly regulated protein domains associated with cerebellum-preferred versus non-cerebellum-preferred isoforms. Domains shown are those above the median frequency. (**F**) (Top) Included and excluded domains in cerebellum, calculated as (inclusion count/total cerebellum) − (exclusion count/total non-cerebellum). (**G**) (Left) Counts of differentially included AFE events by transcript coding category. (Right) Protein sequence similarity between cerebellum and left-ventricle AFE transcript pairs. (**H**) PSI values of AFEs in the ARAP1 gene across heart and brain tissues. (**I**) (Top) Transcripts associated with each AFE in ARAP1; (bottom) InterPro domain architecture of proteins encoded by these transcripts. (**J**) Changes in PPI networks between ARAP1-202 and ARAP1-201 driven by domain differences. (**K**) GO Biological Process enrichment of unique protein interactions enabled by the additional domain in ARAP1-202, generated using the hypeR R package [55].

While all alternative RNA processing types exhibited tissue-specific clustering—particularly between heart, brain, and blood—AFEs displayed the most distinct and robust separation of tissue subtypes (Fig. 6A and Supplementary Fig. S5A). Looking at AFE changes across left-ventricle and cerebellum, we found a significant shift toward inclusion of proximal AFEs in cerebellum (Fig. 6B). We found that 17% of the AFEs preferred in cerebellum resulted in a change in inclusion of NLS motifs—relative to a baseline of 6% of annotated AFE causing shifts in NLS motifs (Fig. 6C). These AFE are also involved in changing signal peptides, transmembrane domains, coiled-coils, and primarily intrinsically disordered regions between the left-ventricle and cerebellum tissues (Fig. 6D). These results align with prior publications discussing the vital role of splicing in localization configurations [2, 77, 78]. The changing AFEs are associated with inclusion of specific protein domains, such as cyclin N-terminal and WD40 domains—both of which are well-established regulators of cerebellar differentiation, proliferation, and development (Fig. 6E and F) [79–81]. A similar pattern emerged in whole blood, where AFE-driven inclusion of domains known to be critical for blood cell maturation and function was consistently observed across comparisons (Supplementary Fig. S5B and C) [82, 83].

Many AFEs across blood, brain, and heart shift transcripts between protein-coding and non-coding forms, while most produce distinct protein isoforms (Fig. 6G). A striking example is the ARAP1 gene, where a shift between an upstream and a downstream AFE is observed in heart versus brain tissues (Fig. 6H). The two resulting transcripts primarily differ in their three most upstream exons, leading to functional differences in the proteins they encode. The cerebellum isoform includes a SAM domain that changes how the protein interacts with others (Fig. 6I), and these interactions are enriched in neuron-related processes like brain development and synapse organization (Fig. 6J and K), highlighting the role of AFE-driven isoform diversity in neural functions. Similar patterns are seen with other processing types—for example, in the *SPEG* gene, which has known heart- and brain-specific isoforms [84]. The left ventricle uses a proximal ALE, while the cerebellum preferentially expresses a distal one (Supplementary Fig. S5D). The brain isoform includes a protein kinase domain (Supplementary Fig. S5E), which may enhance its ability to interact with other proteins. These brain-specific interactions are linked to long-term potentiation, a key process in learning and memory (Supplementary Fig. S5F), showing how ALE-driven isoform diversity contributes to neural signaling.

Together, our results suggest that different types of alternative RNA processing events play distinct roles in shaping protein diversity across human tissues. AFE and ALE are not only the strongest drivers of isoform differences but are also associated with distinct SE inclusion patterns across tissues. AFE leads to the largest relative changes in protein sequence, has the highest rate of rescued frameshifts, while specific proximal AFEs appear to play key roles in cerebellar function. On the other hand, ALE drives the most extensive rewiring of protein interaction networks across human tissues. These findings highlight how tissue-specific splicing strategically leverages different RNA processing events to remodel the functional proteome.

### SpliceImpactR reveals modulated oncogenic PPI in colon adenocarcinoma

**Figure 7.**
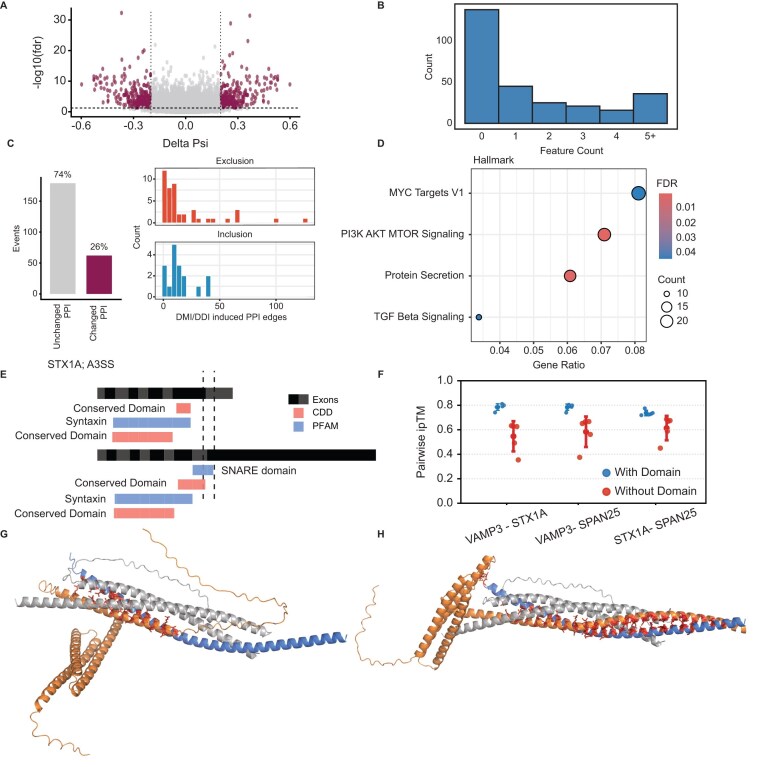
Alternative RNA processing-facilitated PPI changes drive colon adenocarcinoma. (**A**) Differential inclusion results from the application of SpliceImpactR to 23 patients with tumor and normal-matched colon adenocarcinoma samples. Significance of the differential inclusion was determined at FDR < 0.05 and delta PSI > 0.2. (**B**) Distribution of number of protein features changed due to significantly differentially included alternative RNA processing events. Features spanned PFAM, InterPro, SignalP, ELM SLiMs, Mobidb-lite, and CDD. (**C**) Proportion of differentially included events that were found to lead to changed PPIs along with the number of novel interactors with each modulated network. (**D**) MSigDB Hallmark gene set enrichment of the novel interactors from the rewired PPI. Significance determined at FDR < 0.05, generated by ClusterProfiler. (**E**) Protein and feature model of the changed use of the specific A3SS in STX1A that changes in use across tumor and normal samples. (**F**) The change in ipTM between a ColabFold-generated interaction between STX1A, VAMP3, and SNAP25. ColabFold was run using AlphaFold2-multimer_v3 (version 1.5.5), with additional parameter specifications expanded in methods. (**G**) PyMOL-generated visualization of the ColabFold-identified structure and interaction between STX1A, VAMP3, and SNAP25 where the domain-exclusion form of STX1A is used. (**H**) PyMOL-generated visualization of the ColabFold-identified structure and interaction between STX1A, VAMP3, and SNAP25 where the domain-inclusion form of STX1A is used.

We then applied SpliceImpactR to explore the functional consequences of widespread splicing disruptions in colon carcinoma. Analysis of matched tumor and adjacent normal RNA-seq data from TCGA patients identified >600 tumor-associated alternative RNA processing events (ΔPSI > 0.20, FDR < 0.05, Fig. 7A). To validate the AFEs identified by SpliceImpactR, we confirmed that ∼94% of the significantly differentially included AFEs found in colon adenocarcinoma dataset overlap with CAGE peaks from FANTOM5’s [69, 70] set of CAGE data (Supplementary Fig. S6A). The differentially included events reveal widespread remodeling of protein function and interaction networks. Across significant events, ∼26% are predicted to alter PPIs through domain or motif gain/loss (Fig. 7B and C), with the novel interactors enriched in oncogenic pathways including MYC targets and PI3K/AKT/mTOR signaling (Fig. 7D), both central to colon cancer biology. For example, an SE in FGFR2 switches between IIIb and IIIc isoforms, altering an immunoglobulin domain and rewiring interactions with FGF ligands, an established mechanism linked to tumor progression and poor prognosis [85–87].

A striking case involves a tumor-associated A3SS in the STX1A gene that removes part of a SNARE domain (Fig. 7E). This alternative splice site and subsequent domain change has been noted previously in other contexts, such as through the use of IsoformAnalyzer 2.0 in Alzheimer’s disease [66]. SNARE proteins mediate membrane fusion and regulate processes critical for cancer, including trafficking, adhesion, and invasion [88, 89]. Their conserved mechanism involves the assembly of complementary SNARE motifs into a tight bundle that includes VAMP3 and other vesicle-associated SNARE proteins implicated in recycling and retrograde transport [90]. To test whether the loss of a segment in the SNARE domain has any consequence for complex formation, we predicted the STX1A–VAMP3 interaction using a ColabFold-based complex prediction workflow [67, 91]. The interface confidence measured as interface predicted template modeling (ipTM) decreased from 0.79 in the models with domain inclusion to 0.55 in the models without the domain (Fig. 7F), and was accompanied by a decrease in the number of residues involved in the interactions (Fig. 7G and H), indicating that the loss of part of the SNARE domain destabilizes the interaction predictions across all chains.

We then benchmarked SpliceImpactR’s performance across significantly changing transcript usage, protein feature changes, and PPI modulation against tappAS, IsoformSwitchAnalyzeR, and DIGGER. Here, we applied tappAS and IsoformSwitchAnalyzeR to transcript-quantification data from the same set of colon adenocarcinoma tumor and adjacent normal samples. We found that the tools identified similar sets of changes due to alternative splicing, especially at more stringent thresholds (Supplementary Fig. S6B). We also found that SpliceImpactR was able to leverage exon-level inputs to effectively capture transcript swaps due to changes at the 5′ and 3′ ends. We found 22 transcript swaps due to terminal exon events, which were not found by either tappAS or ISAR (Supplementary Fig. S6C). Transcript swaps found by SpliceImpactR due to AFEs, but not found by tappAS or ISAR, are highlighted in *ACSS2, FBP1*, and *SLC44A3* (Supplementary Fig. S6D). Each of these shows a significant change in read coverage of AFE across tumor and normal samples.

We also found alignment in protein consequence results (Supplementary Fig. S6E). This is seen across matches where SpliceImpactR perfectly matches results from tappAS or ISAR and matches where SpliceImpactR’s exon-level focus returns a more conservative subset of protein feature changes across a transcript pair. For validation of the rewired PPI, we compared the predicted impact on interactions in DIGGER for the same set of isoform pairs identified as PPI-altering by SpliceImpactR. We found nearly all isoform pairs showed the same interaction outcomes, with SpliceImpactR showing some changed interactions not reflected by DIGGER (Supplementary Fig. S6F).

Together, our results establish SpliceImpactR as a unified framework for interpreting the functional consequences of alternative RNA processing at exon-level resolution. By integrating protein domains, SLiMs, intrinsically disordered regions, NMD, frameshifts, localization signals, and PPI rewiring within a single pipeline, SpliceImpactR enables mechanistic interpretation of how RNA processing reshapes protein function across tissues and disease states. Applying this framework across human tissues and colon adenocarcinoma reveals that distinct classes of RNA processing events drive specialized functional outcomes, with terminal exon usage emerging as a major regulator of proteome diversity and interaction network remodeling. Beyond recapitulating known biology, SpliceImpactR uncovers previously unrecognized principles linking exon choice to protein function, providing a broadly applicable resource for studying the mechanistic and functional impact of alternative RNA processing in development, physiology, and disease.

## Discussion

SpliceImpactR extends beyond traditional analysis methods by integrating holistic, multi-event analyses to uncover interdependencies and co-regulation among alternative RNA processing events. Unlike previously developed tools such as DIGGER, IsoTV, tappAS, and IsoformSwitchAnalyzeR [24–26, 30, 66, 92], SpliceImpactR offers a streamlined, self-contained, end-to-end framework for downstream analysis following short-read RNA-seq processing and alternative RNA processing event quantification with HIT index and rMATS [37, 38]. SpliceImpactR also requires no external software outside of R for the core functionality, a key distinction from other similar software packages. It also complements other tools—such as the long-read-focused Biosurfer—for comprehensive profiling of isoform diversity [29] by identifying functional consequences of significantly regulated RNA processing events. Moreover, SpliceImpactR uniquely captures the effects of AFE and ALE—the two forms of alternative RNA processing we found to have the most substantial impact. In contrast to prior studies focused on isolated events [7, 9], we reveal patterns of co-regulation across event types and provides a global view of how each RNA processing event type contributes to proteomic diversity. Our large-scale tissue-level analysis using GTEx further demonstrates the coordinated regulation of terminal and internal exon events, underscoring its utility in uncovering biologically meaningful splicing patterns. In line with previous transcriptomic studies, we confirm that SE are the most frequent alternative RNA processing events across human transcripts [7, 93, 94]. We also find extensive alternative splicing-specific PPI patterns and localization signals, consistent with prior literature [2, 4, 77, 78]. However, we found that SEs have a comparatively lower impact on protein diversity. Instead, terminal events exert the most substantial influence on protein sequences, supporting earlier findings that terminal splicing events significantly affect proteomic outcomes [5, 9, 10, 95].

We found intron retention to primarily be associated with changes in identifiable functional protein products in human tissue samples. This is consistent with past observations that mRNAs with RI are often degraded through nonsense-mediated decay due to a PTC. There have been recent advances identifying novel functionality relating to stimuli-response through delayed splicing out. This has been shown to occur as a mechanism for tuning protein expression in settings such as germ cell differentiation and neuronal activity [96–98] and may be related to the RI where no ORF is able to be predicted.

A key discovery of SpliceImpactR involves patterns of frameshift induction and compensatory rescue, expanding on the previously described “snapback” phenomenon identified by Biosurfer [29]. We show that many frame-disrupting events—especially those at the 5′ end—are subsequently corrected by SEs downstream, preserving protein functionality. This supports a pathway in which different RNA processing events may have co-evolved to counterbalance and rescue one another to maintain proteome integrity. The observation of correlated events regulating frameshift and rescue phenomena suggest the potential for coregulation, illuminating the potential for a connection in the development of the alternative processing events. We show this pattern is supported across GTEx tissues and in long-read RNA-seq data.

Our analysis shows that >44% of all annotated alternative RNA processing events are significantly regulated across the 54 human tissues in GTEx. To some extent, it is likely that the isoforms not observed are transiently expressed and quickly degraded. Compared to the full set of annotated events, these tissue-regulated isoforms drive markedly greater changes in protein primary sequence, suggesting a selective regulatory logic that favors isoform usage with the strongest impact on protein structure and function. Among these, AFEs are proportionally more likely to result in protein-altering outcomes relative to the total number of AFEs present in human tissues. Consistent with this, AFEs are more likely to drive larger changes in primary sequence and more dramatic shifts in PSI across tissue. This suggests that AFEs function as a key regulatory axis in tissue-specific gene expression. Further, we found that the domains that AFEs change are enriched for gene pathways related to transcriptional control. We found changing the 3′ end of a transcript has the greatest possibility to change proteins and rewire interactions in the global set of possibilities in annotations, however, in human tissues, we observe that AFE are more often utilized to drive changes.

Domain-level analyses further emphasize the functional consequences of RNA processing, particularly through ALE-mediated changes. Consistent with prior reports on alternative splicing and PPI rewiring [4, 5, 10, 26, 27], we observed that ALEs frequently alter zinc finger domains, while tissue-specific contexts show recurrent inclusion of RNA recognition motifs. In specific tissues, we observed a highly conserved number of protein domains included and excluded due to changing alternative RNA processing events. In cerebellum we identified the consistent inclusion of domains such as WD40 repeats and cyclin N-terminal, both of which are strongly associated with neuronal development and cerebellar function [79, 80], reinforcing the idea that terminal exon usage serves a critical regulatory role in shaping cell-type-specific proteomes. Notably, the coordinated use of AFEs and ALEs in genes like ARAP1 and SPEG between cerebellum and heart tissues provides clear examples of how first and last exon choices drive distinct functional outcomes—further profiling how “functional alloforms”, a term used in Yang *et al*., Cell 2016 to refer to divergently functional isoforms, operate in tissue specific contexts. In these cases, alternative RNA processing choices determine domain presence, protein interaction potential, and tissue-specific roles.

Our application of SpliceImpactR to colon adenocarcinoma data from TCGA provides a case study in how to use the pipeline and the potential discoveries enabled through the pipeline. We found that protein features and PPIs are modulated in tumor samples through alternative RNA processing events, relative to normal-matched samples. The rewired PPI are found in oncogenic genes, further allowing for identifying mechanisms that result in tumor phenotypes, such as displayed in the STX1A–VAMP3 interaction. Further leveraging SpliceImpactR allowed for the identification of clinically relevant protein interactions, such as FGFR–FGF interactions.

## Supplementary Material

gkag827_Supplemental_File

## Data Availability

All scripts are available in the Fiszbein Lab GitHub page (https://github.com/fiszbein-lab/) and on Zenodo (https://doi.org/10.5281/zenodo.21419532). SpliceImpactR is available through Bioconductor’s repository of software: https://bioconductor.org/packages/SpliceImpactR/ and through an R Shiny interface: https://fiszbein-lab.shinyapps.io/SpliceImpactR_Studio/.
