# Supplementary material for: SpliceImpactR maps alternative RNA processing events driving protein functional diversity
Source: Nucleic Acids Res. 2026 Aug 17;54(15):gkag827. doi: 10.1093/nar/gkag827 (PMC13478751; doi:10.1093/nar/gkag827)
Supplement: Supplementary file 1 [file gkag827_supplemental_file.pdf]

SUPPLEMENTAL FIGURES

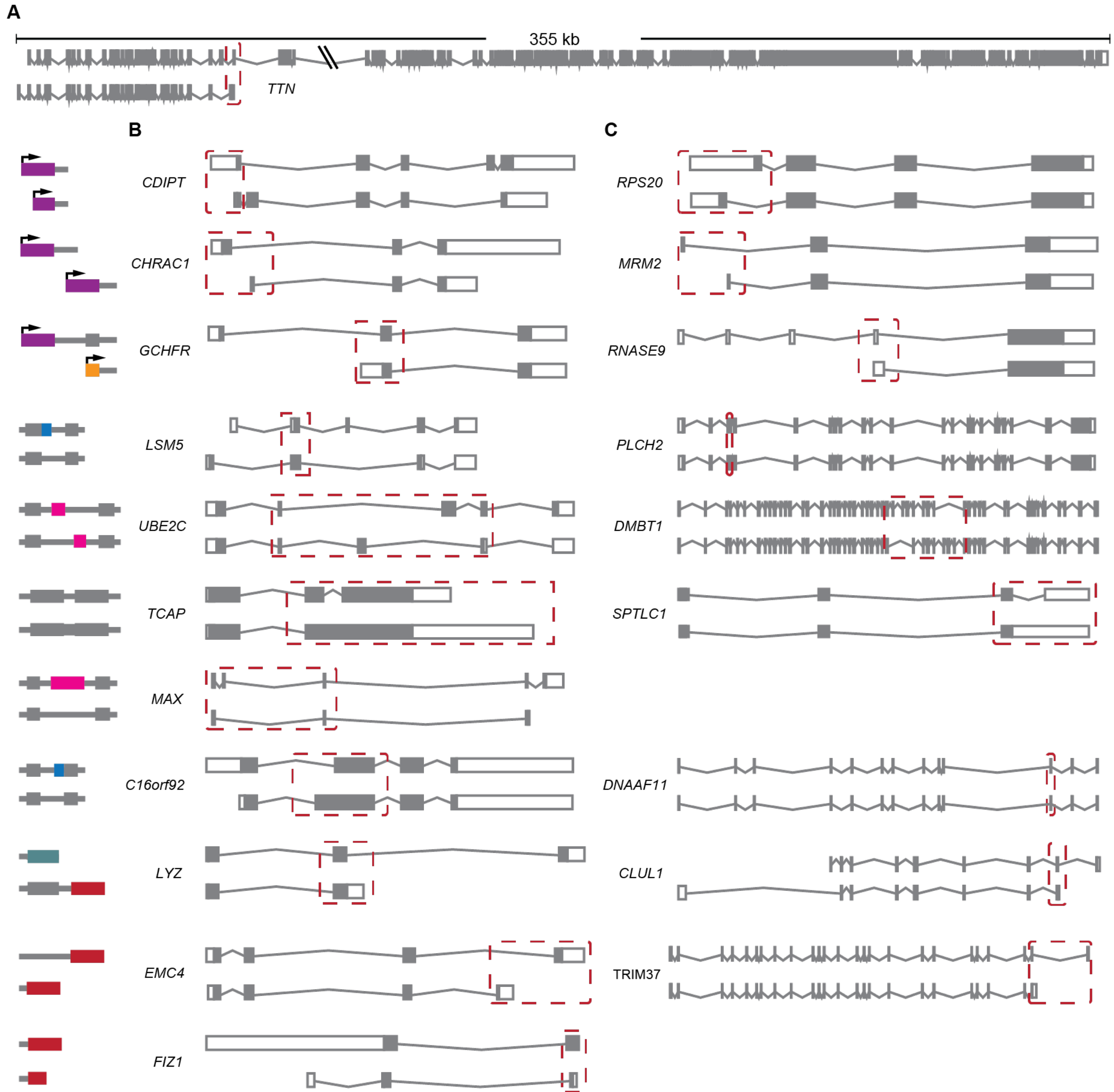

Figure S1. Alternative RNA processing alters transcripts in both expected and unexpected ways, related to Figure 2 **(A)** Example of an Alternative Last Exon (ALE) event in the TTN gene that alters transcript and protein length by more than 98 kb. **(B)** Representative transcript isoform pairs for each major type of alternative RNA processing event, where the event occurs within protein-coding regions and results in changes to the final protein product. **(C)** Representative transcript isoform pairs in which alternative RNA processing events occur within protein-coding regions but do not alter the final protein sequence. No protein-neutral examples were found for aTES. No examples were found that fit this classification for SE or aTES.

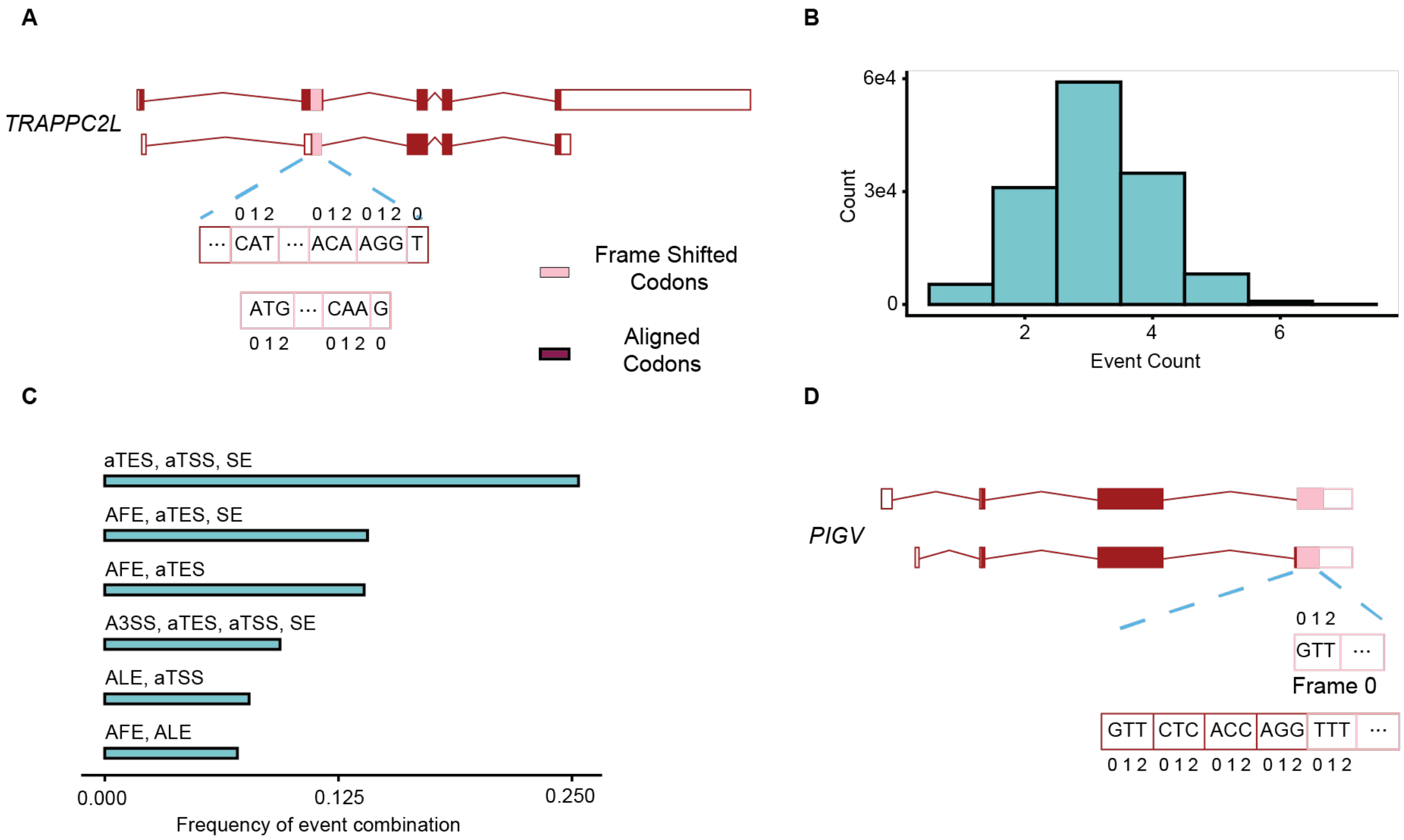

Figure S2. Alternative RNA processing events are co-regulated, related to Figure 3

(A) Example of an in-exon rescue in TRAPPC2L, an A3SS initiates a frame shift and an A5SS rescues the reading frames within the same exon. **(B)** Count of number of co-occurring alternative RNA processing events per transcript pair when at least one event changes. **(C)** Frequency of the top co-occurring event combinations across transcript pairs. **(D)** Example of an A3SS causing a frame shift in just a last exon in PIGV.

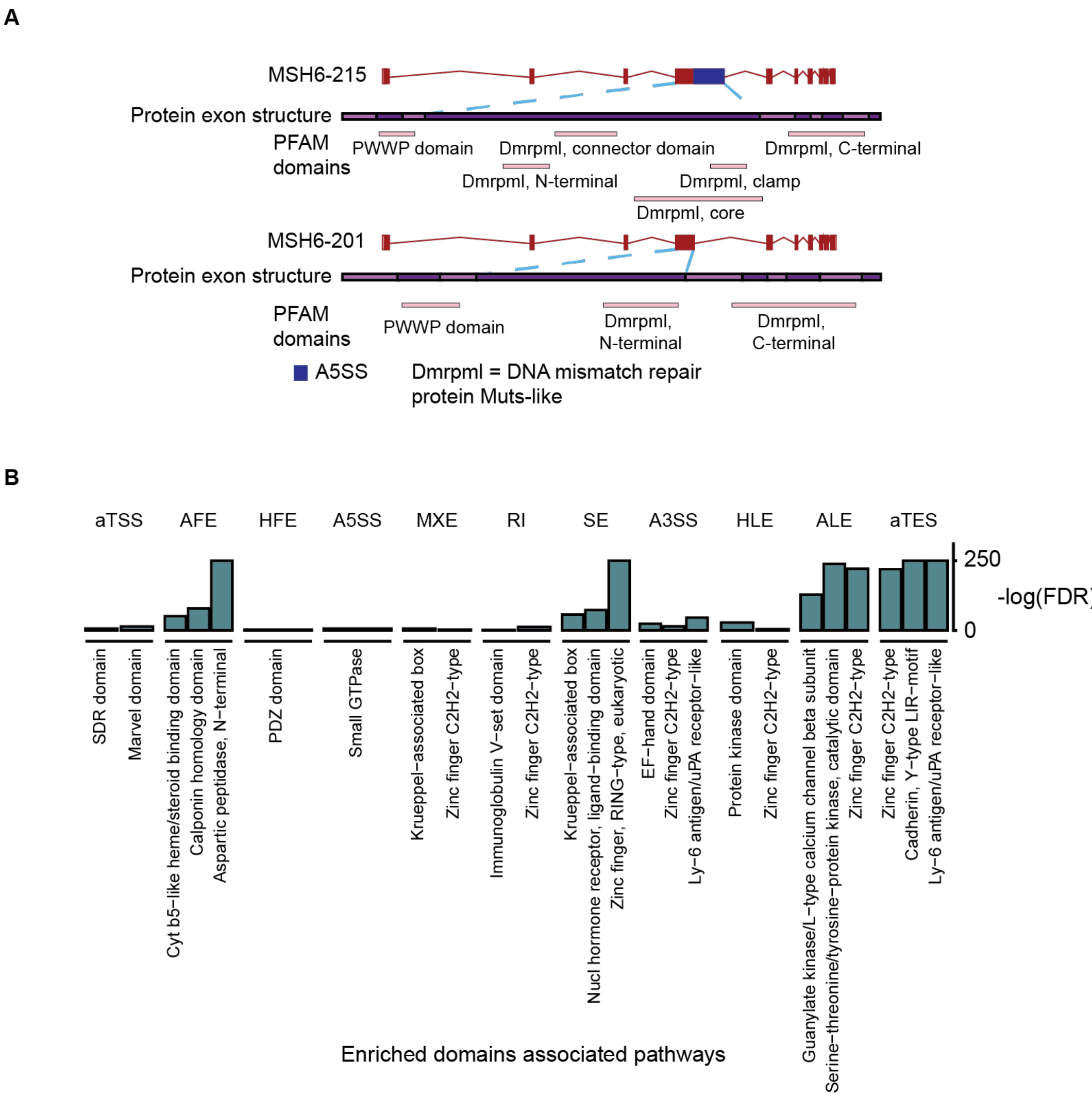

Figure S3. Domain changes due to alternative events drive functional changes, related to Figure 4
(A) Example in MSH6 of multiple DNA mismatch repair protein Muts-like Interpro domains changing due to one A5SS. **(B)** Enriched domains changing in at least 5 genes due to each alternative RNA processing event. Enrichment calculated through a hypergeometric test relative to a background set of all possible domain changes due to alternative RNA processing. P-values were adjusted to generate false discovery rates (FDR < 0.05 considered significant).

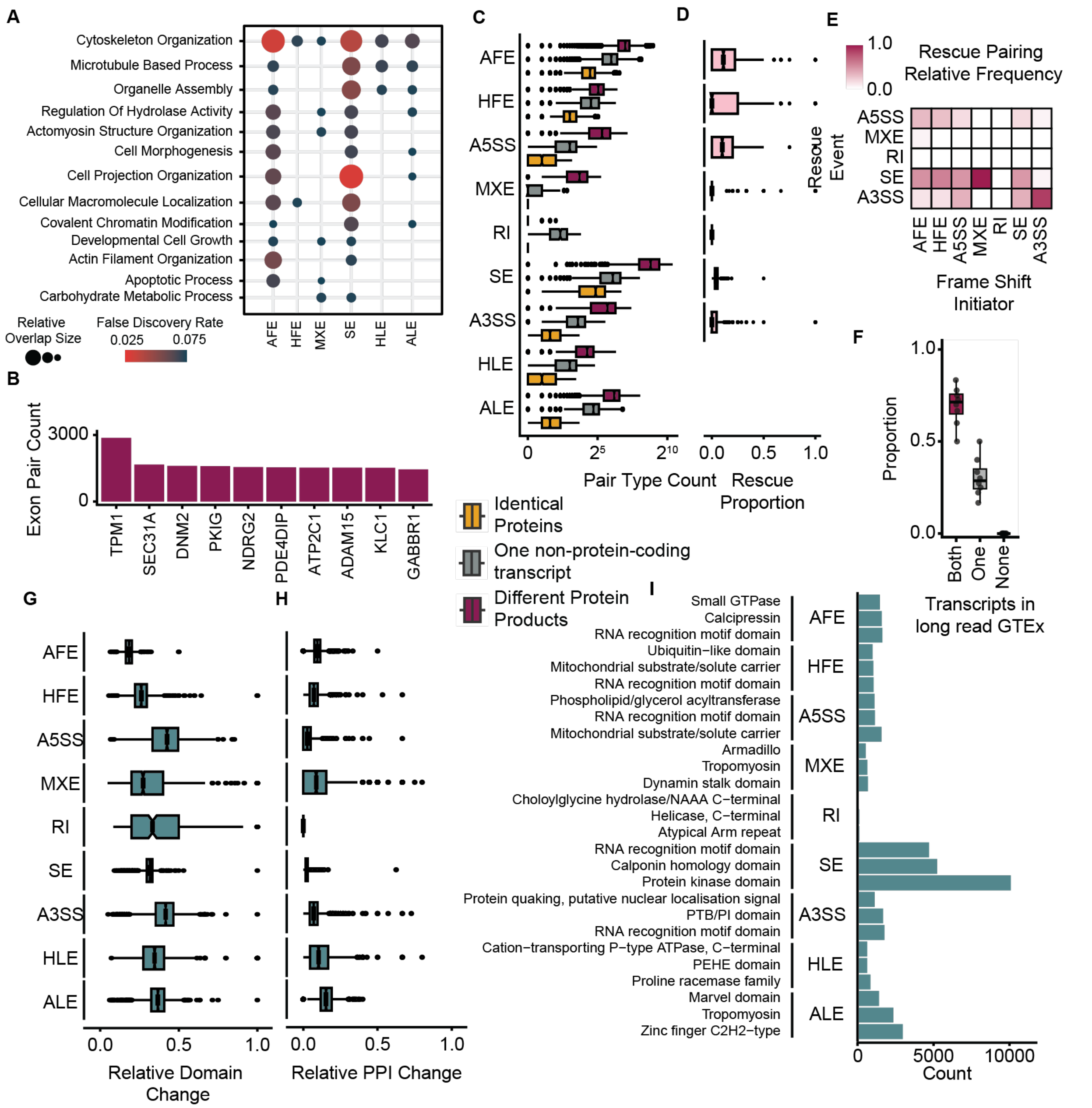

Figure S4. Tissue-specific RNA processing events regulate cell morphogenesis and protein domain inclusion, related to Figure 5 (A) Gene ontology biological process gene set enrichment of genes containing changing alternative RNA processing events across event type. **(B)** Top 10 genes changing across all tissue and event type comparisons. Exon pair count is calculated through summing the number of tissue comparisons across each alternative RNA processing vent a gene contains significantly changing events. **(C)** Distributions of the counts of different protein-coding status of the pairs of transcripts across event type significantly changing across GTEx tissues. **(D)** Proportion of observed frame shifts that are rescued by downstream events in significantly changing events across GTEx tissue. **(E)** Proportion of rescue event types per frameshift event type across all pairwise tissue comparisons. **(F)** Proportion of transcript pairs identified using SpliceImpactR in skeletal muscle comparisons, expressed within long-read GTEx data. Classifications span 'both', 'one', and 'none'. These refer to the number of transcripts from each pair that are represented by the long-read GTEx samples. **(G)** Proportion of events resulting in changes to InterPro domains. A domain is considered "changed" if it overlaps the alternative region and is present in only one isoform. **(H)** Proportion of events resulting in rewired protein–protein interaction (PPI) networks. Rewiring is defined as any change in predicted interactors based on domain presence/absence, using the 3did domain–domain interaction dataset. **(I)** Count of top 3 domains changing due to each alternative RNA processing event.

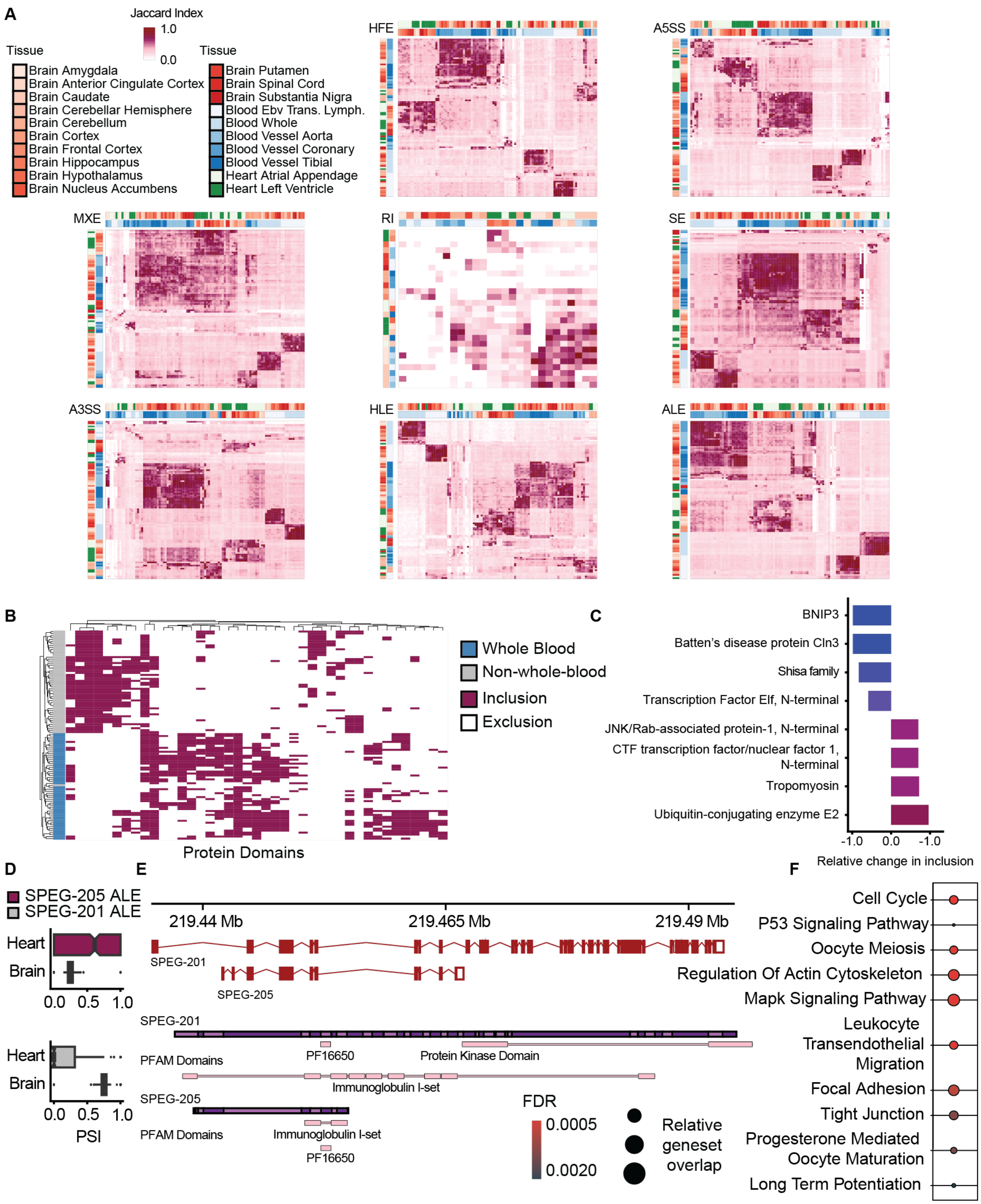

Figure S5. Tissue-specific regulation of alternative events and protein domains, related to Figure 6
(A) Hierarchical clustering using UPGMA on jaccard indices of overlapping genes containing changed alternative events spanning blood, brain, and heart tissues in all alternative RNA processing events other than AFE. **(B)** Domains upregulated in whole blood were assigned to cerebellum and downregulated to non-whole-blood (presence is inclusion of the domain

due to alternative RNA processing). Figure only includes domains present in greater than the median presence number. Hierarchical clustering performed using UPGMA. **(C)** The most regulated protein domains (most included and most excluded) calculated using (count of inclusion / total cerebellum) – (count of exclusion / total non-cerebellum). **(D)** PSI values of SPEG ALE across cerebellum and left-ventricle. **(E)** (top) Transcripts matched to ALE identified, (bottom) proteins and domain composition associated with each matched transcript. **(F)** Gene Ontology Biological Process enrichment of unique PPI due to domain addition in SPEG-201 generated using hypeR.

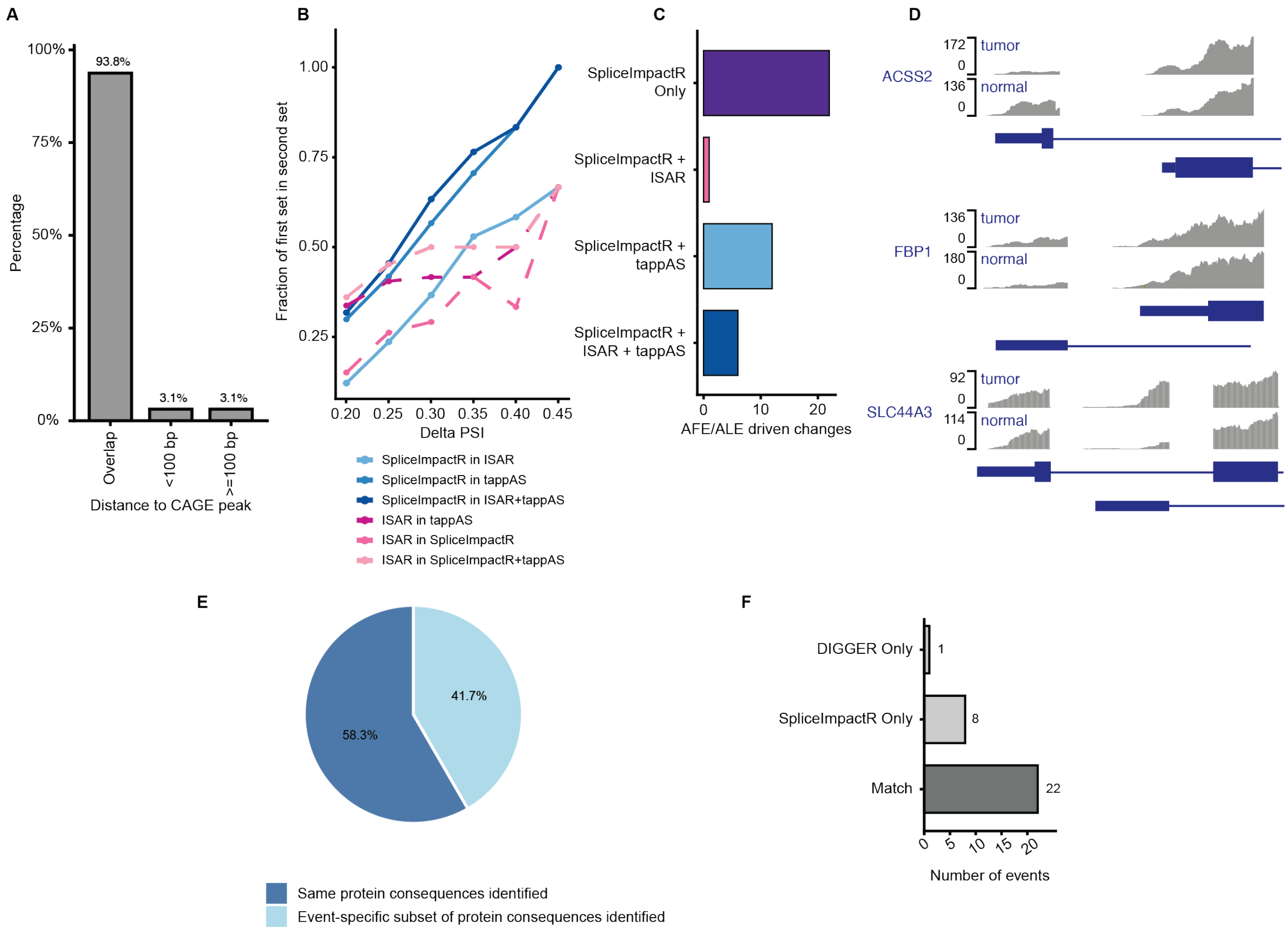

Figure S6. Functional relationship of modulated PPI in colon adenocarcinoma and benchmarking, related to Figure 7
(A) Percentage of overlap between SpliceImpactR-identified significantly differentially included AFE across comparison of colon adenocarcinoma tumor and normal samples and CAGE peaks from FANTOM5 reprocessed peaks. **(B)** Overlap in identified genes with significant transcript swaps due to alterantive splicing across colon adenocarcinoma status between tools at different thresholds (delta PSI sliding from 0.2 to 0.45) across SpliceImpactR, IsoformSwitchAnalyzeR (ISAR), and tappAS. Adjusted p-values set at <0.05 for significance. All three tools were run with their default parameters and protocols. **(C)** Overlap of genes with AFE or ALE driven transcript swaps from SpliceImpactR in tappAS and ISAR. **(D)** Examples where SpliceImpactR identified transcript swaps due to AFE which tappAS and ISAR did nto identify across genes ACSS2, FBP1, and SLC44A3. Tracks are selected from representative samples of colon AD tumor and normal samples. **(E)** Comparison between protein changes in transcript pairs identified as differentially included in at least two of the three tools from colon adenocarcinoma set. Match represents an identical protein change identified, Match (Subset) represents SpliceImpactR identifying a subset of the protein changes identified by other tools as SpliceImpactR investigates on an exon-level and not a transcript level. **(F)** PPI change comparison between SpliceImpactR and DIGGER in colon adenocarcinoma set. Match represents identical PPI changes due to the same protein feature. SpliceImpactR only represents when SpliceImpactR identifies a change that DIGGER has not found. Match (Repeat) represents when SpliceImpactR identifies potential PPI changes due to a change in number of repeat domains that is not represented by DIGGER. Database mismatch represents a true difference in identification of protein domain between two tools/databases. DIGGER Only represents when DIGGER identifies changes that SpliceImpactR does not.
